# The genetics of depression: successful genome-wide association studies introduce new challenges

**DOI:** 10.1038/s41398-019-0450-5

**Published:** 2019-03-15

**Authors:** Johan Ormel, Catharina A. Hartman, Harold Snieder

**Affiliations:** Departments of Epidemiology and Psychiatry, University Medical Center Groningen, University of Groningen, Groningen, The Netherlands

## Abstract

The recent successful genome-wide association studies (GWASs) for depression have yielded more than 80 replicated loci and brought back the excitement that had evaporated during the years of negative GWAS findings. The identified loci provide anchors to explore their relevance for depression, but this comes with new challenges. Using the watershed model of genotype–phenotype relationships as a conceptual aid and recent genetic findings on other complex phenotypes, we discuss why it took so long and identify seven future challenges. The biggest challenge involves the identification of causal mechanisms since GWAS associations merely flag genomic regions without a direct link to underlying biological function. Furthermore, the genetic association with the index phenotype may also be part of a more extensive causal pathway (e.g., from variant to comorbid condition) or be due to indirect influences via intermediate traits located in the causal pathways to the final outcome. This challenge is highly relevant for depression because even its narrow definition of major depressive disorder captures a heterogeneous set of phenotypes which are often measured by even more broadly defined operational definitions consisting of a few questions (minimal phenotyping). Here, Mendelian randomization and future discovery of additional genetic variants for depression and related phenotypes will be of great help. In addition, reduction of phenotypic heterogeneity may also be worthwhile. Other challenges include detecting rare variants, determining the genetic architecture of depression, closing the “heritability gap”, and realizing the potential for personalized treatment. Along the way, we identify pertinent open questions that, when addressed, will advance the field.

## Introduction

Major depressive disorder (MDD, henceforth: depression) is a common mental disorder. In most Western countries, MDD has a 1-year prevalence of ~5%^1,2^ and a lifetime prevalence of ~15%^3^. Depression is in the top three of the leading causes of years lived with disability and a significant contributor to premature mortality due to suicide^4,5^. Twin-based heritability estimates typically fluctuate around 35%. Candidate gene studies have not implicated replicable gene variants^6^ and—until very recently—genome-wide association studies (GWASs) had neither^7,8^.

This long drought without much progress in understanding the genetics of depression has recently ended. The CONVERGE consortium focused on a homogeneous subtype of carefully phenotyped recurrent, severe depression, and identified two genetic loci exceeding genome-wide significance levels^9^. Shortly thereafter, GWASs that used the alternative strategy of increasing sample size while using a more lenient, easy to measure, depression phenotype (i.e., “minimal” phenotyping) have identified additional genetic variants^10–12^, culminating in the most recent successes of GWASs conducted in the UK Biobank (*n* = 322,580; 16 independent loci associated with broad depression phenotypes)^13^, the PGC MDD working group (130,664 MDD cases and 330,470 controls; 44 independent loci)^14^, and the Howard et al. meta-meta-analysis of data on 807,553 individuals (246,363 cases and 561,190 controls) from the three largest GWASs of depression using minimal phenotyping^15^. Howard and colleagues identified 102 independent variants of which 87 replicated in an independent sample. Finally, GWAS findings on multiple psychiatric phenotypes were leveraged to identify eight novel independently replicated depression loci^16^.

With this successful harvest of GWAS loci for depression, the next wave of challenges has come to the fore. Using the watershed model of genotype–phenotype relationships as a conceptual aid and recent genetic insights on other complex traits such as height, we discuss first why it took so long, followed by seven challenges that need further work to advance the field. The biggest challenge is probably the identification of genetic variants that are causally involved in MDD. These challenges are not unique to depression but apply in varying degrees to highly polygenic conditions in general. Nonetheless, we focus this paper on depression for two reasons: (1) Depression is an interesting mental disorder because of its high prevalence and relatively small twin-based heritability, and (2) It affords a clear focus and obviates the need to include information about many other polygenic conditions. Depression contrasts strongly with human height, a complex trait like depression, which is—unlike depression—highly heritable and measured with less error. Below, we often use height as a contrasting complex trait. Gene finding studies for height have shown much more rapid returns than for depression and may give insight into what we might expect for depression in the near future with much larger sample sizes compensating for its lower heritability and larger measurement error.

## Why did it take so long?

**Fig. 1:**
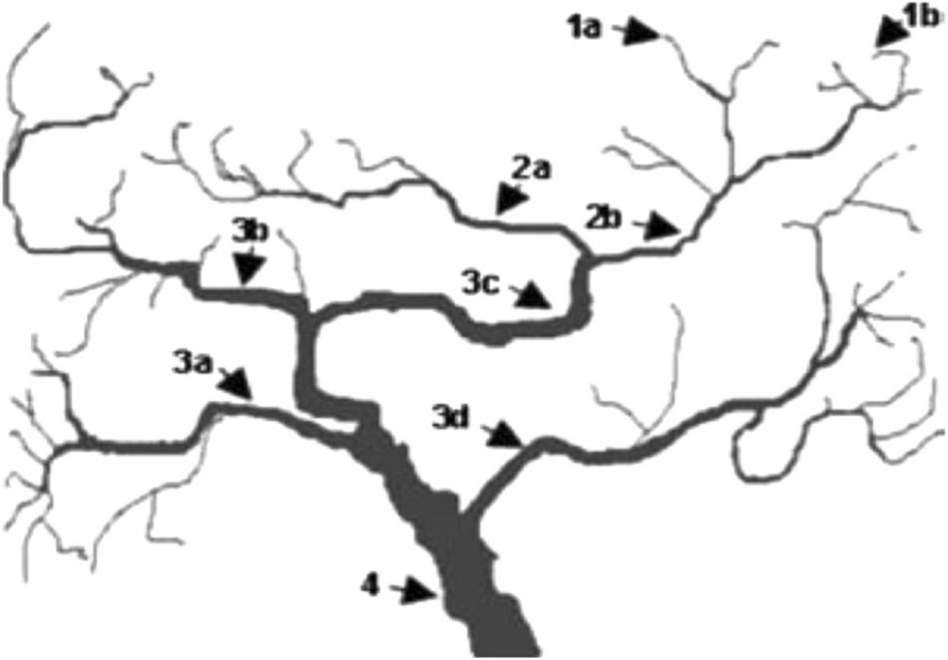
The watershed model of the pathways connecting upstream genetic variants to downstream depression. Mutations at specific loci (1**a**, 1**b**) disrupt narrowly defined mechanisms such as transmission of dopamine in the prefrontal cortex (2**b**). This and other narrowly defined mechanisms contribute noise to more broadly defined mechanisms, such as working memory, facial emotion recognition (3**c**). The latter mechanisms in conjunction with several other mechanisms (3**a**, 3**b**, 3**d**) affect observable phenotypes, such as stress sensitivity, affect regulation. All tributaries eventually flow into fitness (4). Adapted from Keller & Miller, 2006^17^

Keller and Cannon’s watershed analogy of the genotype–phenotype relationship is helpful in explaining why the identification of genetic loci for depression has been extremely difficult (Fig. 1)^17^. Much like the numerous streams of the Amazonian watershed that merge and eventually empty into the Atlantic Ocean, sets of genetic variants influence upstream micro-biological processes (narrowly defined mechanisms; e.g., dopamine transmission) that influence intermediate “downstream” meso-biological processes (e.g., working memory, facial emotion recognition) that in turn affect macro-biological processes (e.g., stress sensitivity, affect regulation), which contribute to the overall fitness of the individual. Gene variants that are directly involved in an upstream process not only affect that upstream process, but indirectly also many processes and traits downstream. If enough noise is present in particular upstream processes, specific behavioral syndromes may arise, such as symptoms of mental disorder. The more upstream (specific, narrow) the (intermediate) phenotype is, the closer its relationship to the genetic variants that affect it, thereby increasing the probability that the associated genetic variants are truly involved in the production of the phenotype.

Depression is a rather downstream phenotype, almost at the level of fitness (Fig. 1). This feature may explain why it took so long for GWASs of depression to become successful. First, as illustrated by the watershed model of genotype–phenotype relationships, multiple tributaries may lead to the same phenotypic outcome (so-called equifinality), with the implication that depression is etiologically heterogeneous, consistent with current insights. This hampers attempts to identify the genetic loci for depression since all individuals with depression are lumped together, which dilutes the explained variance of a particular variant^18^.

Second, depression is not only etiologically but also phenotypically heterogeneous. Approximately 1500 DSM-IV symptom combinations can fulfill the diagnostic criteria^19^. Two patients diagnosed with depression may have very few symptoms in common. In addition, phenotypic heterogeneity will be enhanced by co-occurring psychiatric problems. Two patients having similar depression symptoms may have different comorbid conditions (e.g., anxiety disorder versus substance use disorder). Again, this will dilute the effects of a particular variant.

Third, the upstream processes involved in depression are hardly known, with problematic consequences for the use of “endophenotypes”. To date, the endophenotype approach in depression has not been able to live up to its promises. Many depression-related endophenotypes proposed in the past were based on stress research and neuroimaging, and thus still at relatively low levels in the watershed model. Such endophenotypes tend to be as genetically complex as their outcome phenotypes^20^.

Fourth, phenotypes downstream in the watershed are more likely to be “constructs” rather than biological “entities”, which introduces measurement error. Depression, whether measured by inventories, (semi-)structured interviews, or clinical assessments, is subjective and based on reported and observed affect, cognition, and behavior. Human height, by comparison, is a much simpler phenotype that can be obtained objectively and reliably. Moreover, measurement of depression is complicated by its mixed course, ranging from a single lifetime episode to chronic-recurrent. Even during an episode symptoms often fluctuate. This complicates measurement since retrieving “the most severe episode” from memory implies retrospective assessment with all its reliability problems. For example, it has been shown that SNP heritability is considerably higher for emotional problems when focusing on the stable variance over time compared to single measures per time point, which further illustrates the relative advantage of height, which remains constant over the adult life course, over depression as a phenotype for genetic studies.

## Challenges pertinent to the interpretation and utility of detected genetic variants

With the recent GWAS successes of identifying more than 80 replicated loci, the next wave of challenges for the genetic study of depression come to the fore.

### Challenge 1. Prioritizing likely causal genes for functional follow up

An important characteristic of GWASs is that the identified variants merely flag genomic regions without necessarily providing a direct link to the underlying biological mechanisms^21^. In addition, the identified variant may not be (directly) causal to the phenotype of interest but to other phenotypes that are strongly associated with the phenotype of interest, including comorbid conditions and intermediate traits in the causal pathway leading to the final outcome. Furthermore, effect sizes of individual genetic variants are typically very small (although this does not rule out that effect sizes on currently unknown micro-biological phenotypes higher in the watershed can be large)^22^. All in all, the selection of the most promising signals and the discovery of their functional consequences represent a major challenge. Given the costs and difficulties of conducting functional studies, prioritization of likely causal genes is very important. For the approximately 80+ depression loci this is a formidable task. To date, bioinformatics analyses have been the main strategy.

Fine-mapping of identified loci is typically used as a first step to limit the “credible set of SNPs” that likely include the causal variant(s) responsible for the observed GWAS signals. Transethnic differences in linkage disequilibrium can be used to improve its resolution^23^. However, with the exception of the CONVERGE study of recurrently depressed Han Chinese women, depression GWASs have been limited to individuals of European ancestry. Therefore, more GWASs in other ethnic groups are needed to aid fine mapping efforts. Further bioinformatic post-GWAS follow up analyses leverage the fact that there are only two biological mechanisms that can explain true SNP–phenotype associations: (1) the SNP may alter the amino acid coding (i.e., a nonsynonymous SNP) changing the protein structure and potentially its function, alternatively; (2) it may exert its phenotypic effect through influencing the expression of the gene. Therefore, bioinformatic post-GWAS pipelines will check whether GWAS signals will be in high linkage disequilibrium with nonsynonymous SNPs within nearby genes and use publicly available expression Quantitative Trait Loci (eQTL) resources from relevant tissues such as the brain (GTEx, Braineac) or whole blood to check which SNPs in the identified loci are also associated with gene expression^24^.

Recent tools integrate evidence from GWAS with eQTL data within a Mendelian randomization framework allowing assignment of likely causality of genes within loci^25–27^. The Mendelian randomization approach is based on the fact that the DNA sequence is fixed, which implies that causation can only flow in one direction, allowing the use of genetic markers as instrumental variables. With the advent of GWAS and the recent explosion in variant discovery, this simple idea has been applied to great effect^28^. It has been used in particular to examine the causality of correlated phenotypes and the origins of genetic correlations (see also Challenge 4). But it can also be used to explore the causal role of genes within identified genetic loci for the phenotype of interest^25^.

**Fig. 2:**
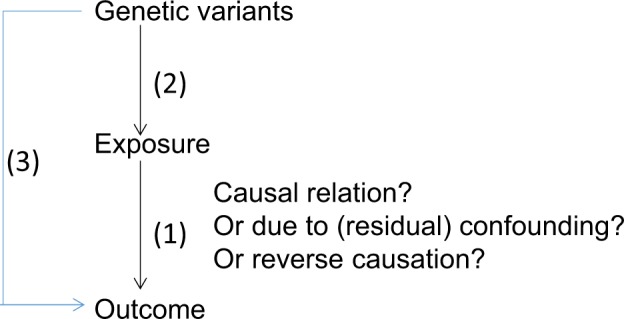
Framework of a Mendelian randomization study in its most basic form. Adapted from Verduijn, 2010^27^

Mendelian randomization in its most basic form is summarized in Fig. 2 where the causal relationship between an exposure (e.g., obesity) and an outcome (e.g., depression) is investigated (association 1 in Fig. 2) using genetic variants known for influencing the exposure (obesity) (association 2) as an instrumental variable, by estimating the association between the genetic variants and the outcome (depression) (association 3)^27^.

**Fig. 3:**
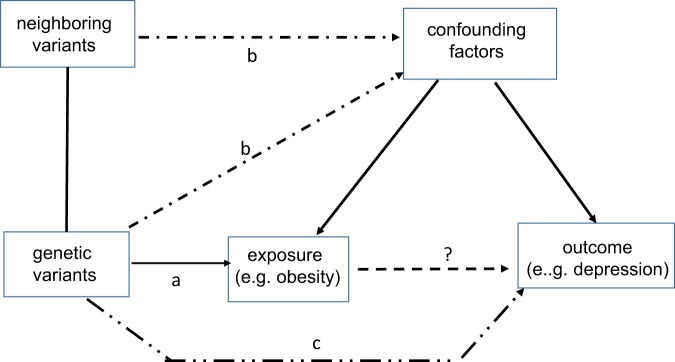
Assumptions made in Mendelian randomization: (**a**) presence of a robust association between genetic variants and exposure (here depression), (**b**) absence of (direct/indirect) association between generic variant and confounding factors, and (**c**) absence of other pathways between genetic variants and outcome. Adapted from Verduijn, 2010^27^

Mendelian randomization has three important assumptions (see Fig. 3). First, the genetic variants should have a robust and strong relationship with the exposure (a). Second, the variants must not be associated with factors that confound the association between exposure and outcome (b). Third, the genetic markers for the exposure only influence the outcome through their effect on the exposure and not through any other pathway (c). Although the latter two assumptions are hard to prove, many Mendelian randomization sensitivity analyses have recently been developed that are less reliant on these assumptions^29^.

Further bioinformatic analyses may explore whether genes within the GWAS loci are preferentially expressed in certain tissues or enriched in certain networks and pathways, and whether these genes are targets of existing (e.g., psychiatric) medications^22,30^. Ultimately, unequivocal evidence of underlying mechanisms will have to come from functional studies such as the one showing a role in synapse pruning for the *C4A* gene in schizophrenia^31^. Developing and increasing throughput of either cell-based or animal model assays to investigate the many GWAS loci for function will be one of the main challenges for the immediate future.

### Challenge 2. Finding rare and more common variants

Given the relationship between sample size and the number of detected loci, it is to be expected that larger sample sizes will identify additional loci. These will include common variants with (even) small(er) effects and probably rare variants of moderate-to-large effect although their role in depression is currently unknown^32^. The brief history of GWASs of, for example, schizophrenia, supports the expectation that with larger sample sizes rare variants can be discovered. In general, the contribution of rare variations of strong effect tends to be larger for early-onset, highly heritable, severe (e.g., neurodevelopmental disorders, including schizophrenia) disorders and lesser for disorders that are less heritable, less severe and with a later onset, such as depression^30^. But that does not exclude a role for rare variants in depression.

The support that rare variants may play a role in depression also comes from large GWASs of complex traits outside psychiatry, most notably human height^33,34^. Some rare variants (minor allele frequencies [MAFs] 0.8–2.1%) had large effects, implicating a 2 cm difference in height. The explained variance of genetic variants is a simple function of both effect size and MAF. As such, despite their much larger effect size, the rare height-associated genetic variants each explain, on average, similar amounts of variation at the population level as common variants. The much lower effect size of common variants is “compensated” by their much higher frequency.

Fortunately, costly whole genome or exome sequencing may not be necessary to find rare variants, as large GWAS sample sizes of a million individuals or more, imputed to very large sequenced reference samples will offer sufficient resolution and power in the low frequency range and are now increasingly feasible^35,36^. Furthermore, the strong phenotypic and genetic correlation between depression and other mental disorders may be leveraged to improve power and identify both additional common and novel rare variants for depression^16^.

### Challenge 3. Establishing the genetic architecture of depression

Initially, GWASs of complex traits operated from the simple common disease–common variant (CDCV) model, positing that a moderate number (<100) of gene variants of intermediate frequency (MAF > 5%) with small-to-moderate effect (OR > 1.5) account for the heritability of the trait. The results of recent GWAS studies of complex traits, including height, have shown this CDCV model to be wrong for most types of complex traits^37–39^. Four alternative models of genetic architecture have been proposed: (i) the *infinitesimal* model assuming that heritability is due to a large number (»100) of small-effect common variants, (ii) the *rare allele* model, assuming that heritability is due to a large number of rare variants with relatively large-effects (including copy number variants), (iii) the *broad-sense heritability* model, assuming that in addition to additive effects of common variants, heritability is due to rare variants, non-additive GxG (dominance, epistasis) and GxE interactions as well as epigenetic effects, and (iv) the *omnigenic* model hypothesizing that the genetic architecture of complex traits is characterized by very large numbers of peripheral, more general genes and a limited number of “core” genes, assumed to be more disease specific^37,40^.

Apart from the yet to be detected occasional rare variants, the infinitesimal model may provide a good approximation for a highly polygenic disorder like depression. Regarding non-additive GxG and GxE interactions, decisive evidence is lacking as to date GWASs have not been designed and powered to detect GxG and GxE interactions, neither for depression, nor for other mental disorders or height. Recent studies were unable to show a sizable influence of dominance for a wide range of complex traits^41^. Previous attempts to study GxE on the basis of single candidate genes^42,43^ are now being replaced by the use of polygenic risk scores (e.g., refs. ^44,45^). which may offer more promise for detection of GxE interaction, although the polygenic risk scores will only provide a general measure of genetic susceptibility for depression that does not directly indicate underlying mechanisms. Moreover, the use of polygenic risk scores neglect the possibility that the impact of GxE interaction is not consistent across different genes or pathways; some genetic pathways may show GxE interaction but others may not or in the reverse direction. Nonetheless, it is likely that these interaction studies will eventually be successful given, for example, “indirect” evidence of substantial GxE interactions between personality traits and environmental exposures^46,47^. Regarding the omnigenic model, Wray and colleagues have recently argued that, although intuitively appealing, there is insufficient empirical evidence for the existence of its hypothesized core genes^18^.

Irrespective of the correct model for the genetic architecture, it must reserve an honorable seat for environmental influences. In addition to the aforementioned interactions, two epidemiological observations support a substantial environmental component in the etiology of depression: Although depression can be found everywhere, substantial national and regional variations in prevalence exist^3,48^. Because of the ultimately subjective nature of the measurement process, these prevalence differences are hard to interpret. Furthermore, strong effects on risk for depression have been documented for long-term difficulties (e.g., taking care of a dementing partner, persistent unemployment, victim of chronic bullying) which in addition moderate the depression risk of stressful life events (e.g., acute illness of child, let down by friend)^46,49–51^. The latter short-term factors are typically thought to play a role in the timing of depression onsets pushing susceptible individuals across the diagnostic threshold. Although such environmental effects partly reflect depression’s genetic background (i.e., gene–environment correlation); they are nonetheless likely to have an extra, additive, contribution to, as well as interaction with, genetic risk in explaining the depression phenotype.

### Challenge 4. Genetic pleiotropy and unraveling causal relationships with other traits

Pleiotropy is the phenomenon whereby a genetic variant influences two or more phenotypes^52^. In line with high comorbidity across mental disorders, GWAS findings also indicate substantial genetic overlap, although the extent thereof was perhaps unexpected^53^. SNP-based genetic correlations (*r*_*g*_) between depression and other mental disorders are substantial^14^. Pleiotropy is not specific to psychiatry and has also been shown between depression and somatic conditions^14,54,55^.

Part of the genetic correlations may derive from symptom/diagnostic overlap, comorbidity or can even be an artefact of diagnostic misclassification. It is useful to make a distinction between heterogeneity due to different symptom patterns that all meet MDD’s diagnostic criteria and heterogeneity due to comorbidity with other mental disorders, a common phenomenon. Genetic correlations may also reflect a common cause. For example, virtually all mental disorders involve sensitivity to stressful situations which is why individual differences in appraisal of and coping with stressful experiences have an impact on the severity of their manifestation. This commonality implies that genetic variants that influence appraisal and coping may turn up in GWASs of these mental disorders, although they are at best causally involved in a generic way, not strictly part of the specific disorder’s pathophysiology. In the watershed model, these shared appraisal-coping or executive functioning related variants are probably located relatively upstream, with many confluences downstream. The higher hierarchical position makes it “easier” to be involved in multiple phenotypes than variants more downstream.

A unique feature of genetic as opposed to classic epidemiological associations that aids in unraveling causality is that confounders (or third variables) that influence both genetic markers and outcome phenotypes do not exist (DNA is fixed). Mendelian randomization can thus also be used to disentangle the causes of correlated traits using genetic markers to distinguish between alternative causal explanations such as reverse causation and shared causes^56^. One important requirement for effective Mendelian randomization is the availability of a sufficient number of genetic markers associated with the exposure as their combined effect determine the strength of the instrumental variable. This means that distinguishing causality between depression and associated traits using Mendelian randomization has only recently become possible. The continued discovery of additional genetic variants for depression will be important to improve their strength as an instrumental variable and hence the power of Mendelian randomization analyses in order to distinguish between alternative causal pathways, thereby providing fresh clues to old questions.

### Challenge 5. Closing the “heritability gap”

Genetic variants detected by GWAS typically explain only a fraction of the total family- or twin-based heritability. This has become known as the missing heritability problem or heritability gap^41,57^. Recently introduced methodology now also allows calculation of the SNP- or chip-based heritability (*h*^2^_SNP_), which is the proportion of phenotypic variance jointly accounted for by all variants on a standard GWAS chip. *h*^2^_SNP_ provides an upper bound estimate of the genetic effects that could be detected by a (well-powered) GWAS^58^. The remainder is likely due to rarer and structural variants that have until recently not been captured by regular GWAS arrays^35^. The latest PGC depression GWAS estimates this *h*^2^_SNP_ at ~9%, which is only around a quarter of the heritability based on twin and family studies of ~35%. However, measurement error and heterogeneity in phenotype definition between the different PGC cohorts may explain part of the difference in heritability as the CONVERGE study found a *h*^2^_SNP_ between 20 and 29% within their cohort of carefully assessed women with early-onset recurrent depression.

Another part of the heritability gap may be attributed to potentially inflated twin heritability estimates caused by gene by common (C) (or shared) environment (GxC) interactions^45^. That is, genetic effects depend on environmental factors shared by twins that grow up in the same family but not by unrelated individuals in the GWAS samples. The statistical models used in twin studies to estimate twin-based heritability fully attribute the joint effect (GxC) to the genetic component, thus inflating heritability estimates and reducing the contribution of shared environment. The GxC explanation “solves” not only (part of) the heritability gap but the shared environment paradox as well. This paradox refers to the apparent inconsistency that, in contrast to epidemiological studies, the statistical models used in twin studies typically find hardly any shared-environmental variance while many (distal) environmental risk factors are shared by twins in the same family, e.g., poverty, family instability, child neglect, neighborhood stressors, minority status, SES^59^. As pointed out by Uher and Zwicker^45^, this paradox disappears if we realize that the impact of these shared-environmental factors depends on characteristics shared to a larger extent by monozygotic than dizygotic twins, i.e., genetic variants.

The difference between heritability explained by GWAS identified variants and the *h*^2^_SNP_ has also been more aptly termed “hidden” heritability as future larger GWASs are expected to detect additional signals still hidden in the noise. Furthermore, new generations of denser and better-imputed GWAS arrays are expected to capture more rare and structural variants, which will increase the *h*^2^_SNP_ and decrease its gap with the total (potentially inflated) (family and twin-based) heritability^35,57^.

### Challenge 6. Reducing the phenotypic heterogeneity

A major challenge is the identification of genetic variants that are causally involved in MDD and this is aggravated by the use of minimal phenotyping (based on a few symptoms), which, as recently shown, may have yielded cases unrepresentative of MDD that are enriched by people “with non-specific sub-clinical depressive symptoms and depression secondary to a comorbid disease”^60^.

Traditionally, endophenotypes of depression have been used in the hope that this would shorten the distance between genes and phenotype, and consequently reduce genetic heterogeneity. To date, the expectations have not come true as the used endophenotypes were genetically not less complex. However, the recently identified genetic signals may provide new insights into underlying pathophysiological pathways and networks providing clues on more suitable less complex endophenotypes (micro-biological phenotypes) that are at higher levels in the watershed model and thus closer to the genes. Constructs assessed through task performance such as reward sensitivity and attentional biases may have potential utility as endophenotypes if they are associated with genetic signals. Given their transdiagnostic relevance, they may also account for other disorders in which these mechanisms are active as well.

Some argue that another way forward lies in the reduction of phenotypic heterogeneity, by targeting a specific subtype such as early-onset recurrent melancholia or a specific symptom cluster^9^. Others believe that a broader phenotype approach will provide a more tractable target for genetic studies, as this identifies more signals^11^. Findings of the CONVERGE consortium based on a sample size that was much smaller than the other recent depression GWASs suggests that in-depth phenotyping may pay off^9^. In addition to high-quality measurement of depression, this study focused on recurrent severe depression which may be genetically more homogeneous. However, CONVERGE differed in additional aspects from the other MDD GWASs, including the Chinese sample, the focus on women, and analytical approach. We cannot rule out that these differences contributed to CONVERGE’s success.

The recent GWASs successes with broad phenotypes of depression and neuroticism^61^ seem to support the broad trawl approach^13^. But note that phenotype broadness is subject to more noise because the likelihood that identified loci are not involved in the physiology of the phenotype of interest increases with broadness (see Challenge 4).

In contrast to broader milder depressive states, other clinically recognizable “subtypes” of depression including early-onset recurrent depression and the more severe subtypes of melancholic, bipolar, and psychotic depression may also be less heterogeneous^62–64^. It is also important to keep in mind that even highly homogeneous subtypes defined by behavioral symptoms are still placed low in the watershed model and remain multifactorial, with multiple underlying etiological pathways although they may be less genetically heterogeneous than all “depressions” lumped together.

Novel approaches improving the phenotype definition of depression may be needed. We outline two complementary approaches below that make use of depression measured at the most narrow level of individual symptoms.

#### Bottom-up: individual symptoms of depression as a starting point

Symptom-specific GWASs to examine their genetic background may be an interesting next step. Previous depression GWASs have used composite scores or diagnostic case-control designs. Based on data from the UK Biobank, Nagel and colleagues^65^ showed that the composite score of neuroticism, an important personality trait that partially overlaps with depression^66^, directs the focus to genetic variants that affect the majority of aggregated items, i.e., “global variants”. The genetic signal of “local” variants, affecting only one or a few of the aggregated items, was severely diluted^65^. Given its multidimensional and heterogeneous nature, it is plausible that symptom-specific GWASs of depression will yield similar findings. Some initial evidence comes from a relatively small sample showing that *h*^2^_SNP_ of four depression symptom components (appetite, depressed mood and anhedonia, insomnia, and anxiety) was different, suggesting the possible merit of more narrowly defined phenotypes^67^.

#### Top-down: use hierarchical dimensional models

Hierarchical dimensional models such as the Hierarchical Taxonomy of Psychopathology (HiTOP)^68^ show how psychopathology dimensions can be arranged in a hierarchy, ranging from very broad “spectrum level” dimensions (e.g., distress, thought disorder, disinhibited externalizing, etc.), to more specific clusters of symptoms. For instance, the distress spectrum, one of the five internalizing spectra consists of the lower clusters (sub-dimensions) of irritability, anhedonia, numbing, physical panic, suicidality, dysphoria, retardation, lassitude, appetite loss, insomnia, and generalized anxiety^69^. These models hypothesize a hierarchy linking spectrum level dimensions with highly pleiotropic variants which subsume lower order symptom clusters with less pleiotropic variant clusters. This phenotypic ordering from narrow to broad may help in the interpretation of genetic findings.

### Challenge 7. Personalized treatment

An important issue is the utility of identified variants for individualized treatment of depression. Three applications may be genomic (polygenic) risk prediction, genome editing, and the identification of novel “druggable” targets, respectively. GWAS results can be used for genomic risk prediction. Increased risk could prompt more intensive surveillance or even prophylactic treatments unrelated to a specific causal mechanism (cf. preventive mastectomy in case of high genetic breast cancer risk). The widespread pleiotropy among mental disorder phenotypes (genetic correlations) can be used to improve genomic risk prediction, so that this might benefit personalized medicine^70^. However, genetic risk-driven prophylactic treatment is not realistic yet, given the low effect size of SNP-based genetic predictors^70^.

Genome-editing technology such as CRISPR/Cas9 may make it possible to change or disable genes in living cells in a precise, cheap, and fast way by cutting, replacing, or adding pieces from the DNA^71,72^. However, it is highly doubtful whether this will ever have relevance for “fixing” depression-associated genetic variants due to their small effect sizes, unknown individual relevance (from population to individual), causal relevance (too upstream), and unwanted “side” effects of genome-editing (genetic pleiotropy).

With regard to pharmacotherapy, virtually all currently used drugs in psychiatry have their origins in chance findings in the previous century, while rational approaches to develop new pharmacological treatments have mostly not paid off. The recent findings from the PGC depression GWAS indicated that lead SNPs in some loci were within genes known to play a role in neuronal development, synaptic function, transmembrane adhesion complexes, and/or regulation of gene expression in brain. In addition, genes that are targets of antidepressant medications were strongly enriched for depression-associated signals, which may indicate pharmacotherapeutic relevance^73^. In addition, some identified loci were associated with clinical features of depression including early-onset, recurrence, and severity, and implicated prefrontal and anterior cingulate cortex in the pathophysiology of depression (brain regions showing MRI anatomical and functional differences between MDD cases and controls). Thus, current genomic findings may have substantial potential for the development of new depression medications. The future will tell.

However, the complex genetic nature of depression raises the question for whom drugs developed on the basis of GWAS findings will work. For a highly complex trait-like depression, each individual probably carries a unique combination of protective and risk alleles (see ref. ^18^ for an illustration). The more polygenic a trait, the more combinations of these sets of alleles are possible, implying that each individual is likely to have a different combination, including affected individuals whose symptom levels have crossed the diagnostic threshold. This explains why most mental disorders are highly heritable but only weakly inheritable^22^. Due to genetic recombination, the probability that a child will inherit a mix of alleles from an affected parent resulting in a genetic risk sufficiently high to also pass the diagnostic threshold remains fairly small. Thus, effect sizes derived from GWASs of a genetic variant with depression should be interpreted in the context of an averaged background; in individual carriers the contribution of a certain variant may be much larger. A particular drug can be effective only in the subgroup of individuals that share the genetic variant and pathway targeted by the drug. The effectiveness of a drug in the individual case thus depends on the number of possible combinations in which the particular variant is a necessary component to become depressogenic. Ultimately, precision medicine for highly polygenic disorders like depression may depend on successfully matching these unique individual genomic profiles to drug treatments^18^.

## Conclusion

Clearly, the excitement for investigating the genetic background of depression has returned. Prospects to unravel the pathogenesis and etiology of depression and rationally develop pharmacotherapies are better than ever. Now that depression-associated genetic variants have been found, the next wave of challenges has taken center stage. It is likely that larger samples will identify additional common variants and the first batch of rare variants, which will reveal the genetic architecture of depression, its subtypes and broader phenotypes. It is also likely that taking common (C) (or shared) environment (GxC) interactions into account will contribute to unearthing part of the missing heritability^45^. The GxC explanation not only bridges (part of) the heritability gap but also may explain the shared environment paradox.

The largest challenges will be to identify the causal variants of depression itself and to determine which variants merely correlate with the depression phenotype because they are causally involved in its determinants. In addition to bioinformatics, Mendelian randomization may help to resolve this causal web and will become increasingly effective with the identification of additional genetic variants of depression and related phenotypes. To date, Mendelian randomization has almost exclusively been used to aid in the interpretation of genetic correlations between correlated phenotypes, but it may also assist in distinguishing causal from pleiotropic depression variants. Another approach to clarify the causal status of genetic variants might be comparing markers associated with narrowly (e.g., early-onset recurrent melancholic depression) versus broadly defined depression phenotypes. Likewise, comparing the variants associated with different levels of the “depression hierarchy” (symptoms, clusters of symptoms, internalizing dimensions, general psychopathology factor) might be informative. Finally, the fact that highly polygenic traits are only weakly inheritable despite substantial heritability^22^ could explain the seeming paradox that antidepressants may benefit individual patients enormously despite their modest average efficacy on a group level compared to pill-placebo^74^.

## References

[1] Steel Z (2014). The global prevalence of common mental disorders: a systematic review and meta-analysis 1980–2013. Int. J. Epidemiol..

[2] Ferrari AJ (2013). Burden of depressive disorders by country, sex, age, and year: findings from the global burden of disease study 2010. PLoS Med..

[3] Bromet E (2011). Cross-national epidemiology of DSM-IV major depressive episode. BMC Med..

[4] Erskine HE (2015). A heavy burden on young minds: the global burden of mental and substance use disorders in children and youth. Psychol. Med..

[5] Whiteford HA, Ferrari AJ, Degenhardt L, Feigin V, Vos T (2015). The global burden of mental, neurological and substance use disorders: an analysis from the global burden of disease study 2010. PLoS ONE.

[6] Bosker FJ (2011). Poor replication of candidate genes for major depressive disorder using genome-wide assoiation data. Mol. Psychiatry.

[7] Wray, N. R. et al. Genome-wide association study of major depressive disorder: new results, meta-analysis, and lessons learned. *Mol. Psychiatry* **17**, 36–48 (2012). 10.1038/mp.2010.109 PMC3252611 21042317

[8] Sullivan, P. & 96 Psychiatric Genetics Investigators. Don’t give up on GWAS. *Mol. Psychiatry* **17**, 2–3 (2012).

[9] Cai N (2015). Sparse whole-genome sequencing identifies two loci for major depressive disorder. Nature.

[10] Okbay A (2016). Genetic variants associated with subjective well-being, depressive symptoms, and neuroticism identified through genome-wide analyses. Nat. Genet..

[11] Hyde CL (2016). Identification of 15 genetic loci associated with risk of major depression in individuals of european descent. Nat. Genet..

[12] Direk N (2017). An analysis of two genome-wide association meta-analyses identifies a new locus for broad depression phenotype. Biol. Psychiatry.

[13] Howard DM (2018). Genome-wide association study of depression phenotypes in UK Biobank identifies variants in excitatory synaptic pathways. Nat. Commun..

[14] Wray NR (2018). Genome-wide association analyses identify 44 risk variants and refine the genetic architecture of major depression. Nat. Genet..

[15] Howard, D. M. Genome-wide meta-analysis of depression in 807,553 individuals identifies 102 independent variants with replication in a further 1,507,153 individuals. *Nat. N**eurosci.* **22**, 343–352 (2019). 10.1038/s41593-018-0326-7 PMC6522363 30718901

[16] Amare, A. T. et al. Bivariate genome-wide association analyses of the broad depression phenotype combined with major depressive disorder, bipolar disorder or schizophrenia reveal eight novel genetic loci for depression. *Mol. Psychiatry*10.1038/s41380-018-0336-6 (2019). 10.1038/s41380-018-0336-6 PMC7303007 30626913

[17] Keller MC, Miller G (2006). Resolving the paradox of common, harmful, heritable mental disorders: which evolutionary genetic models work best?. Behav. Brain Sci..

[18] Wray NR, Wijmenga C, Sullivan PF, Yang J, Visscher PM (2018). Common disease is more complex than implied by the core gene omnigenic model. Cell.

[19] Ostergaard SD, Jensen SOW, Bech P (2011). The heterogeneity of the depressive syndrome: when numbers get serious. Acta Psychiatr. Scand..

[20] Franke B (2016). Genetic influences on schizophrenia and subcortical brain volumes: large-scale proof of concept. Nat. Neurosci..

[21] Vaez A (2015). In silico post genome-wide association studies analysis of C-reactive protein loci suggests an important role for interferons. Circ. Cardiovasc. Genet..

[22] Visscher PM (2017). 10 Years of GWAS discovery: biology, function, and translation. Am. J. Hum. Genet..

[23] Franceschini N (2012). Discovery and fine mapping of serum protein loci through transethnic meta-analysis. Am. J. Hum. Genet..

[24] Westra H (2015). Cell specific eQTL analysis without sorting cells. PLoS Genet..

[25] Zhu Z (2016). Integration of summary data from GWAS and eQTL studies predicts complex trait gene targets. Nat. Genet..

[26] Prins, B. et al. Investigating the causal relationship of C-reactive protein with 32 complex somatic and psychiatric outcomes: a large-scale cross-consortium mendelian randomization study. *PLoS Med.* **13**, e1001976 (2016). 10.1371/journal.pmed.1001976 PMC4915710 27327646

[27] Verduijn M, Siegerink B, Jager KJ, Zoccali C, Dekker FW (2010). Mendelian randomization: use of genetics to enable causal inference in observational studies. Nephrol. Dial. Transplant..

[28] Lawlor DA, Harbord RM, Sterne JAC, Timpson N, Smith GD (2008). Mendelian randomization: using genes as instruments for making causal inferences in epidemiology. Stat. Med..

[29] Hemani G, Bowden J, Smith GD (2018). Evaluating the potential role of pleiotropy in mendelian randomization studies. Hum. Mol. Genet..

[30] Sullivan PF (2018). Psychiatric genomics: an update and an agenda. Am. J. Psychiatry.

[31] Sekar A (2016). Schizophrenia risk from complex variation of complement component 4. Nature.

[32] Peterson RE (2017). The genetic architecture of major depressive disorder in Han Chinese women. JAMA Psychiatry.

[33] Wood AR (2014). Defining the role of common variation in the genomic and biological architecture of adult human height. Nat. Genet..

[34] Marouli E (2017). Rare and low-frequency coding variants alter human adult height. Nature.

[35] Yang J (2015). Genetic variance estimation with imputed variants finds negligible missing heritability for human height and body mass index. Nat. Genet..

[36] Evangelou E (2018). Genetic analysis of over 1 million people identifies 535 new loci associated with blood pressure traits. Nat. Genet..

[37] Gibson G (2012). Rare and common variants: twenty arguments. Nat. Rev. Genet..

[38] Gratten J, Wray NR, Keller MC, Visscher PM (2014). Large-scale genomics unveils the genetic architecture of psychiatric disorders. Nat. Neurosci..

[39] Uher R (2009). The role of genetic variation in the causation of mental illness: an evolution-informed framework. Mol. Psychiatry.

[40] Boyle EA, Li YI, Pritchard JK (2017). An expanded view of complex traits: from polygenic to omnigenic. Cell.

[41] Nolte IM (2017). Missing heritability: is the gap closing? An analysis of 32 complex traits in the lifelines cohort study. Eur. J. Hum. Genet..

[42] Culverhouse RC (2018). Collaborative meta-analysis finds no evidence of a strong interaction between stress and 5-HTTLPR genotype contributing to the development of depression. Mol. Psychiatry.

[43] Gerritsen L (2017). HPA axis genes, and their interaction with childhood maltreatment, are related to cortisol levels and stress-related phenotypes. Neuropsychopharmacology.

[44] Peyrot WJ (2014). Effects of polygenic risk scores on depression in childhood trauma. Br. J. Psychiatry.

[45] Uher R, Zwicker A (2017). Etiology in psychiatry: embracing the reality of poly-gene-environmental causation of mental illness. World Psychiatry.

[46] Ormel J, Oldehinkel AJ, Brilman EI (2001). The interplay and etiological continuity of neuroticism, difficulties and life events in the etiology of major and subsyndromal, first and recurrent depressive episodes in later life. Am. J. Psychiatry.

[47] Caspi A, Hariri AR, Holmes A, Uher R, Moffitt TE (2010). Genetic sensitivity to the environment: the case of the serotonin transporter gene and its implications for studying complex diseases and traits. Am. J. Psychiatry.

[48] Weissman MM (1996). Cross-national epidemiology of major depression and bipolar disorder. JAMA.

[49] Bifulco A, Brown GW, Moran P, Ball C, Campbell C (1998). Predicting depression in women: the role of past and present vulnerability. Psychol. Med..

[50] Brilman EI, Ormel J (2001). Life events, difficulties and onset of depressive episodes in later life. Psychol. Med..

[51] Brown GW (1998). Genetic and population perspectives on life events and depression. Soc. Psychiatry Psychiatr. Epidemiol..

[52] Gratten J, Visscher PM (2016). Genetic pleiotropy in complex traits and diseases: implications for genomic medicine. Genome Med..

[53] Smoller JW (2018). Psychiatric genetics and the structure of psychopathology. Mol. Psychiatry.

[54] Amare AT, Schubert KO, Klingler-Hoffmann M, Cohen-Woods S, Baune BT (2017). The genetic overlap between mood disorders and cardiometabolic diseases: a systematic review of genome wide and candidate gene studies. Transl. Psychiatry.

[55] Milaneschi Y (2017). Genetic association of major depression with atypical features and obesity-related immunometabolic dysregulations. JAMA Psychiatry.

[56] van den Oord EJCG, Snieder H (2002). Including measured genotypes in statistical models to study the interplay of multiple factors affecting complex traits. Behav. Genet..

[57] Tropf FC (2017). Hidden heritability due to heterogeneity across seven populations. Nat. Human. Behav..

[58] Yang J (2010). Common SNPs explain a large proportion of the heritability for human height. Nat. Genet..

[59] Polderman TJC (2015). Meta-analysis of the heritability of human traits based on fifty years of twin studies. Nat. Genet..

[60] Cai, N., Kendler, K. & Flint, J. Minimal phenotyping yields GWAS hits of low specificity for major depression. *BioRxiv*10.1101/440735 (2018).

[61] Luciano M (2018). Association analysis in over 329,000 individuals identifies 116 independent variants influencing neuroticism. Nat. Genet..

[62] Lamers F (2013). Evidence for a differential role of HPA-axis function, inflammation and metabolic syndrome in melancholic versus atypical depression. Mol. Psychiatry.

[63] Parker G, Paterson A, Hadzi-Pavlovic D (2015). Cleaving depressive diseases from depressive disorders and non-clinical states. Acta Psychiatr. Scand..

[64] Wakefield JC, Schmitz MF (2014). Predictive validation of single-episode uncomplicated depression as a benign subtype of unipolar major depression. Acta Psychiatr. Scand..

[65] Nagel M, Watanabe K, Stringer S, Posthuma D, van der Sluis S (2018). Item-level analyses reveal genetic heterogeneity in neuroticism. Nat. Commun..

[66] Ormel J (2013). Neuroticism and common mental disorders: meaning and utility of a complex relationship. Clin. Psychol. Rev..

[67] Pearson R (2016). Additive genetic contribution to symptom dimensions in major depressive disorder. J. Abnorm. Psychol..

[68] Kotov R (2017). The hierarchical taxonomy of psychopathology (HiTOP): a dimensional alternative to traditional nosologies. J. Abnorm. Psychol..

[69] Waszczuk MA, Kotov R, Ruggero C, Gamez W, Watson D (2017). Hierarchical structure of emotional disorders: from individual symptoms to the spectrum. J. Abnorm. Psychol..

[70] Maier RM (2018). Improving genetic prediction by leveraging genetic correlations among human diseases and traits. Nat. Commun..

[71] Doudna JA, Charpentier E (2014). The new frontier of genome engineering with CRISPR-Cas9. Science.

[72] Zhang F, Wen Y, Guo X (2014). CRISPR/Cas9 for genome editing: progress, implications and challenges. Hum. Mol. Genet..

[73] Kathiresan S (2015). Developing medicines that mimic the natural successes of the human genome lessons from NPC1L1, HMGCR, PCSK9, APOC3, and CETP. J. Am. Coll. Cardiol..

[74] Cipriani A (2018). Comparative efficacy and acceptability of 21 antidepressant drugs for the acute treatment of adults with major depressive disorder: a systematic review and network meta-analysis. Lancet.

